# Effect Of G2706A and G1051A polymorphisms of the ABCA1 gene on the lipid, oxidative stress and homocystein levels in Turkish patients with polycystıc ovary syndrome

**DOI:** 10.1186/1476-511X-10-193

**Published:** 2011-10-28

**Authors:** Muammer Karadeniz, Mehmet Erdoğan, Zengi Ayhan, Murat Yalcın, Murat Olukman, Sevki Cetinkalp, Gulinnaz E Alper, Zuhal Eroglu, Asli Tetik, Vildan Cetintas, Ahmet G Ozgen, Fusun Saygılı, Candeger Yılmaz

**Affiliations:** 1 Department of Endocrinology, Ege University School of Medicine, Izmir, Turkey; 2 Department of Biochemistry, Ege University School of Medicine, Izmir, Turkey; 3 Department of Medical Biology, Ege University School of Medicine, Izmir, Turkey; 4 Ege University Hospital, Ege University School of Medicine, Department of Pharmacology, Izmir, Turkey; 5 Division of Internal Medicine, Sifa University, Health Application and Research Center, Izmir, Turkey; 6 Department of Endocrinology, Sifa University, Health Application and Research Center, Izmir, Turkey

## Abstract

**Background:**

Obesity, insulin resistance and hyperandrogenism, crucial parameters of Polycystic ovary syndrome (PCOS) play significant pathophysiological roles in lipidemic aberrations associated within the syndrome. Parts of the metabolic syndrome (low HDL and insulin resistance) appeared to facilitate the association between PCOS and coronary artery disease, independently of obesity. ABCA1 gene polymorphism may be altered this components in PCOS patients.

In this study, we studied 98 PCOS patients and 93 healthy controls. All subjects underwent venous blood drawing for complete hormonal assays, lipid profile, glucose, insulin, malondialdehyde, nitric oxide, disulfide levels and ABCA genetic study.

**Results:**

In PCOS group fasting glucose, DHEAS, 17-OHP, free testosterone, total-cholesterol, triglyceride, LDL-cholesterol and fibrinogen were significantly different compare to controls. The genotype ABCA G2706A distribution differed between the control group (GG 60.7%, GA 32.1%, AA 7.1%) and the PCOS patients (GG 8.7%, GA 8.7%, AA 76.8%). The frequency of the A allele (ABCAG2706A) was higher in PCOS patients than control group with 13,0% and 23,2%, respectively. In this study, the homocystein and insulin levels were significantly higher in PCOS patients with ABCA G1051A mutant genotype than those with heterozygote and wild genotypes.

**Conclusions:**

We found higher percentage of AA genotype and A allele of ABCA G2706A in PCOS patients compare to controls. The fasting insulin and homocystein levels were significantly higher in PCOS patients with ABCA G1051A mutant genotype than those with heterozygote and wild genotypes.

## Introduction

Polycystic ovary syndrome (PCOS) is one of the most common endocrine diseases and typically presents with chronic anovulation and hyperandrogenism [1]. PCOS is a common endocrine disorder, affecting between 4% and 8% of the women of reproductive age [2]. PCOS usually arises during puberty and is marked by hyperinsulinemia and hyperandrogenism. Adolescents with PCOS have an increased risk of developing health problems later on in life such as type 2 diabetes, cardiovascular disease, and infertility [3]. Studies suggest that PCOS is associated with increased risk of coronary heart disease (CHD) [4]. Elevated plasma Hcy levels and oxidative stress parameters are considered as an independent risk factor for CVD [5-7].

Dyslipidemia is possibly the most common metabolic abnormality of PCOS, although the findings of relevant studies have been variable and a substantial percentage of women with PCOS might still have normal lipid profiles [8]. Qualitative alterations of lipoproteins have also been described in PCOS [9].

Obesity, IR and hyperandrogenism, crucial parameters of PCOS play significant pathophysiological roles in lipidemic aberrations associated with the syndrome. IR represents another key factor implicated in the dyslipidemia of PCOS. Glueck et al. reported that 46% of women with PCOS suffered from the metabolic syndrome. Within this subgroup of women, lipid abnormalities were found to be extremely common. 95% of these women demonstrated decreased levels of high-density-lipoprotein (HDL), whereas 56% had hypertriglyceridemia [10].

Lean women with PCOS were shown to have decreased HDL and HDL_2 _levels when compared to women with normal ovarian function, whereas obese PCOS women also had elevated triglyceride levels [11].

Hyperlipidemia was frequently connected with the disorders in apolipoproteins, receptors, enzymes, or cofactors in proteins related to lipoprotein metabolism. The changes happened in ABCA1 gene can play an important role amongst these changes. ABCA1 gene was considered to be responsible for the cause of Tangier disease [12].

Luciani and collagues were defined ABC transporter family for the first time in 1994. ATP-binding cassette (ABC) genes encode a large family of transmembrane proteins. These proteins bind ATP in order to control the transition of different molecules (such as cholesterol) from cell membranes [13].

This study aims at exploring the association between metabolic, oxidative stress parameters (including malondialdehyde, (MDA) nitric oxide (NO) and disulfide levels (SH)) and demographic parameters and ABCA1 gene polymorphisms in polycystic ovary syndrome patients.

## Materials and methods

### Patients

In this study, we studied 98 PCOS patients and 93 healthy controls. All researchs with human samples were done with written informed consent of the patients and with approval of the ethical committee of the Ege Universty Hospital. The patients had been referred to the Endocrinology and Metabolism Disease outpatient clinic at the Ege University Hospital. PCOS was defined by the Rotterdam PCOS consensus criteria [14]. Patients who had DM, hyperprolactinemia, congenital adrenal hyperplasia (diagnosed with the adrenocorticotropic hormone stimulation test), thyroid disorders, Cushing's syndrome, hypertension, hepatic or renal dysfunction were excluded from the study. Confounding medications, including oral contraceptive agents, hypertensive medications and insulin-sensitizing drugs, and those which may affect the metabolic criteria were questioned. Another 93 healthy young volunteer females matched for age, body mass index (BMI), and allele frequency were included from the study, and considered as the control group. Their health state was determined by medical history, physical and pelvic examination, and complete blood chemistry. The patients with PCOS and the control group were genetically unrelated.

### Study protocol

At study entry, all subjects underwent venous blood drawing for complete hormonal assays, lipid profile, glucose, insulin and ABCA genetic study. All blood samples were obtained in the morning between 08.00 and 09.00 hours after an overnight fasting, and resting in bed during early follicular phase of the spontaneous or P-induced menstrual cycle. During the same visit, all subjects underwent anthropometric measurements including BMI and detail history, systolic and diastolic blood pressure. In present study, data related to the serum malondialdehyde, nitric oxide and disulfide levels, homocystein and fibrinogen levels and the genetic evaluation of ABCA will be shown and discussed.

### Biochemical assay

Serum total cholesterol, LDL and HDL cholesterol were measured by Olympus AU 2700 automated analyzer. Plasma insulin concentrations were determined by Immunolite 2000 using two-site chemiluminescent immunometric assay.

### Methods for plasma MDA, NO, total sulfhydryl group measurements

All reagents were purchased from Sigma and Merck. MDA was determined by a modified spectrophometric method of Yagi K [15] using tetrametoxypropan as Standard and BioTek MicroQuant microplate reader. NO was determined by measuring stable NO end-products-nitrite and nitrate levels using Miranda's spectrophometric method [16], while total sulfhydryl groups was measured using Ellman's reagent by Sedlak and Lindsay's method [17].

### Measurement of plasma homocysteine

Venous blood samples were centrifuged at 1000× g for 10 min and the serums were stored at -80°C until the analysis -not exceeding three months. In this study, serum total homocysteine levels were measured by Fluorescence Polarization Immunoassay Method (IMX Homocysteine Assay, Abbott Diagnostics No: 7D29-20). Dilution method was used for those whose serum homocysteine levels were higher than 50 μmol/L. All samples were prepared as 200 μl. Three controls were used in each sample for calibration of the device. For each of the controls, the results including ranges were accepted as low control (5, 25-8, 75), medium control (10.0-15.0), and high control (20.0-30.0) μmol/ml.

### Genetic Analysis

DNA isolations were carried out by using High Pure PCR Template Preparation Kit (Roche Applied Science, Germany) from peripheral blood samples of control and study group cases taken to tubes with EDTA.

For ABCA 1 G1051A polymorphism analysis, by using Forward Primer: 5'-CTC CAA AAGACT TCA AGG ACC C-3', Reverse Primer: 5'-GGC CCA AAA GTC TGA AAG AAC AC-3' primer pair, a DNA fragment of 433 base pairs is amplified by PCR method. For each sample, 25 μl of prepared PCR reaction mixture contains 16, 75 μl of sterile distilled water, 0,5 μl of Forward primer (100 p μl), 0,5 μl of Reverse primer (100 p μl), 2,5 μl Mg^+2 ^(25 mM), 2 μl of dNTP mixture (2 mM), 0,25 μl of Taq DNA polymerase (5 U/μl), and 2,5 μl of genomic DNA.

### i - PCR amplification

Denaturation is carried out at 95°C for 2 minutes, amplification at 94°C for 30 seconds as 35 cycles, 1 minute at 72°C, prolongation at 72°C for 7 minutes by applying PCR protocol.

For ABCA1 G2706A polymorphism analysis, DNA fragment of 350 base pairs is amplified by using Forward Primer: 5'-CAA GTG AGT GCT TGG GAT TG-3', Reverse Primer: 5'-TGC TTT TAT TCA GGG ACT CCA-3' primer pairs by PCR method. For each sample, 25 μl of PCR reaction mixture contains 17, 25 μl of sterile distilled water, 0,5 μl of Forward primer (100 p μl), 0,5 μl of Reverse primer (100 p μl), 2,5 μl Mg^+2 ^(25 mM), 1,5 μl of dNTP mixture (2 mM), 0,25 μl of Taq DNA polymerase (5 U/μl), and 2,5 μl of genomic DNA.

### ii - PCR amplification

Denaturation is carried out for ten minutes at 94°, amplification for 30 seconds at 94°C as 40 cycles, for 45 seconds at 52°C, 1 minutes at 72°C, prolongation for 7 minutes at 72°C by applying PCR protocol. The size of the products obtained after PCR is controlled at gel electrophoresis. PCR products made for the analysis of ABCA 1 G1051A and G2706A gene polymorphisms are evaluated in agarose gel of 2%.

G1051A polymorphism analysis of -ABCA 1 gene is carried out at 37°C for 6 hours by incubating RFLP reaction mixture of 30 μl containing 17 μl of distilled water, 10 μl of PCR product, 2 μl of Buffer O, and 1 μl of EcoO109I enzyme.

G2706A polymorphism analysis of -ABCA 1 gene is carried out by incubating at 30°C for 3 hours the mixture of RFLP reaction of 30 μl containing 17 μl of distilled water, 10 μl of PCR product, 2 μl of Buffer O, and 1 μl of BsaAI enzyme.

Genotyping is carried out after having conducted and displayed the products obtained as a result of enzyme cut for G1051A polymorphism analysis of ABCA 1 gene in agarose gel of 2% and in agarose gel of 3% for G2706A polymorphism analysis.

**Figure 1:**
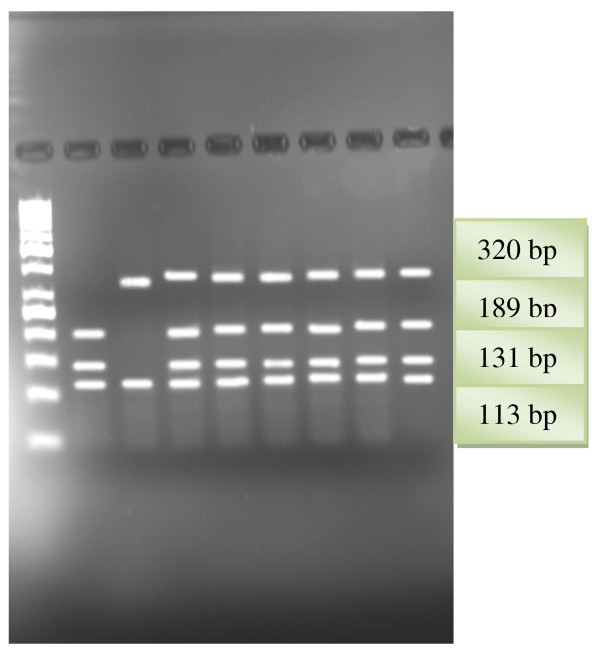
**RFLP patterns of ABCA1 G1051A gene obtained with StyI restriction endonuclease**. Lane1. 50 bp DNA ladder (Fermentas); Lane2. GG genotype; Lane3. AA genotype; Lanes 4,5,6,7,8,9. GA genotypes.

**Figure 2:**
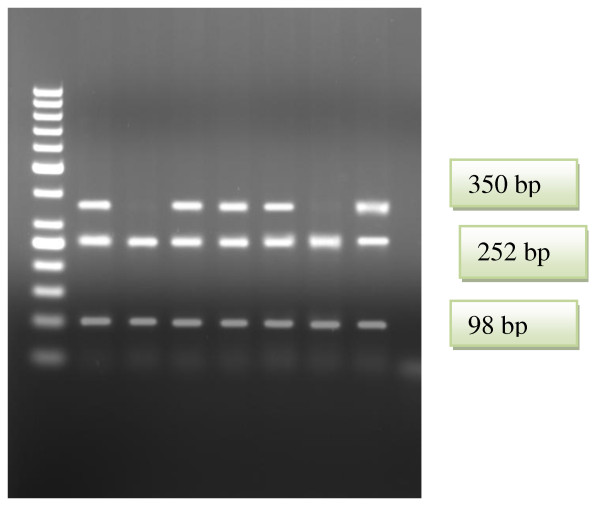
**RFLP patterns of ABCA1 G2706A gene obtained with BasaI restriction endonuclease**. Lane1. 50 bp DNA ladder (Fermentas); Lanes 2, 4,5,6,8 GA genotypes; Lanes 3, 7 GG genotypes.

As a result of G1051A polymorphism analysis, DNA fragments of sizes of 189 base pairs, 131 base pairs, and 113 base pairs in cases of wild genotype, 320 base pairs, 189 base pairs, 131 and 113 base pairs in heterozygote cases, and 320 base pairs and 113 base pairs in mutant cases are obtained (Figure 1). As a result of G2706A polymorphism analysis, DNA fragments of sizes of 252 base pairs and 98 base pairs in cases of wild genotype, 350 base pairs, 252 base pairs and 98 base pairs in heterozygote cases, and 350 base pairs in mutant cases are obtained (Figure 2).

## Results

### I-Demographic, metabolic and oxidative stress parameters of patients and controls

The demographic, hormonal and metabolic parameters of the PCOS and control groups are shown in Table 1. In the PCOS group fasting glucose, DHEAS, 17-OHP, free testosterone, total-cholesterol, triglyceride, LDL-cholesterol and fibrinogen were significantly (P < 0.05) different in comparison with healthy women (Table 1). The fasting glucose levels were significantly (P < 0.05) higher in PCOS than in control women, whereas no difference was observed in fasting insulin concentrations between the groups (Table 1). No significant differences were detected in age, weight, BMI, homocystein, MDA, NO, SH, estradiol, DHEAS, prolactin, HDL-cholesterol levels between two groups (Table 1).

**Table 1:** Clinical characteristics of patients and controls

	Control Group		PCOS Group		p
	**Mean ± SD**		**Mean ± SD**		

**Age (years)**	25,63	7,67	24,44	5,69	0,567

**Weight**	60,43	15,37	66,17	16,08	0,099

**BMI (k/m^2^)**	24,25	7,68	24,80	5,88	0,436

**HCY μmol/ml**.	10,90	3,60	12,37	4,05	0,073

**MDA (nMol/ml)**	6,47	3,63	5,59	2,55	0,396

**NO (microMol/L)**	8,73	3,04	9,54	4,16	0,595

**SH (mMol/L)**	6,96	6,07	10,92	7,76	0,172

**TAFI**	9,89	5,06	12,14	10,01	0,563

**Fasting Glucose (mg/dl)**	84,39	18,27	91,79	8,99	0,005

**Fasting insulin (mIU/ml)**	11,09	21,22	15,78	33,23	0,507

**Homocysteine (μmol/L)**	1,27	1,24	3,83	9,17	0,168

**Estradiol (pg/ml)**	51,65	58,40	35,13	26,82	0,052

**DHEA-S (μg/dl)**	272,93	131,36	217,19	115,69	0,035

**17 -OHP (ng/ml)**	1,10	1,05	1,85	1,04	0,002

**Total-Testosterone (ng/ml)**	0,92	1,30	0,96	1,85	0,905

**Free-Testosterone (pg/ml)**	1,76	0,83	3,28	2,12	0,001

**Prolactin (pmol)**	17,09	10,76	17,01	8,00	0,969

**Total-cholesterol (mg/dl)**	171,34	61,74	198,95	41,19	0,009

**Triglycerides (mg/dl)**	88,52	50,83	124,00	69,35	0,014

**HDL-C (mg/dl)**	59,37	16,39	57,06	15,39	0,514

**LDL-C (mg/dl)**	98,89	31,83	117,68	31,71	0,01

**Fibrinogen (mg/dl)**	295,85	102,07	373,83	110,84	0,004

### II-Allelic distributions and ABCA genotype frequencies in patients

The frequency of ABCA G1051A and G2706A polymorphism are shown in Table 2. The allelic distribution of ABCA genotypes was in Hardy-Weinberg equilibrium for both groups of women.

**Table 2:** Distribution of ABCA haplotypes and genotypes

*Polymorphism*	*Genotypes/Haplotypes*	*Control* *Group* *n = 93*	*PCOS* *Group* *n = 98*	OR*	95% CI*	P*
**G1051A**	GG (Wild type)	34 36,6%	30 31,2%	R		0.585
	
	GA (Heterozygote)	49 52,7%	53 53,8%	0,836	0,444-1,574	0,579
	
	AA (Mutant)	10 10,8%	15 15,1%	0,609	0,235-1,577	0,307
	
	G	117 62,9%	114 58,1%	0.817	0.538-1.238	0.396
	
	A	69 37,1%	82 41,9%			

***G2706A***	GG (Wild type)	55 60,7%	80 82,6%	R		0.004
	
	GA (Heterozygote)	30 32,1%	9 8,7%	5,029	1,922-13,160	0.001
	
	AA (Mutant)	8 7,1%	9 8,7%	1,118	0,339-3,685	0.855
	
	G	162 87,0%	150 76,8%	2.016	1.094-3.714	0.027
	
	A	24 13,0%	46 23,2%			

The genotype ABCA G1051A distribution didn't differ between the control group (GG 31.2%, AG 53.8%, AA 15.1%) and the PCOS patients (GG 36.6%, AG 52.7%, AA 10.8%) (P > 0.05) The genotype ABCA G2706A distribution differed between the control group (GG 60.7%, GA 32.1%, AA 7.1%) and the PCOS patients (GG 8.7%, GA 8.7%, AA 76.8%) (P < 0.05). The frequency of the polymorphic A allele (ABCA G2706A) was higher in PCOS patients than control group with 13,0% and 23,2%, respectively (p = 0.027, OR:2.016; Table 2).

### III-Effects of ABCA genotypes on lipid profile, demographic and other metabolic parameters in patients

There was no statistically significant difference in PCOS patients between ABCA G1051A genotypes (AA, GA and GG) and BMI, fasting insulin, fasting glucose, triglyceride levels, HDL levels, LDL levels, fasting blood glucose levels, f-testosterone, fibrinogen and 17-OHP levels (p > 0.05; Table 3). The fasting insulin level was significantly higher in PCOS patients with ABCA G1051A mutant genotype than those with heterozygote and wild genotypes (Table 3). No statistically meaningful difference was determined between polymorphism and lipid and other parameter levels in patients carrying ABCA1 G2706A polymorphism (Table 4).

**Table 3:** Biochemical and Hormonal parameters between ABCA G1051A genotypes in patient group

	G1051A Genotype	Mean	SD	95% CI	p
BMI (k/m^2^)	GG	23,64	5,11	21,62-25,66	0,353
	
	GA	25,45	6,386	23,46-27,44	
	
	AA	26,3	6,277	21,81-30,79	

Fasting Glucose (mg/dl)	GG	91,63	6,264	89,15-94,11	0,291
	
	GA	91,79	7,537	89,44-94,13	
	
	AA	96,09	14,883	86,09-106,09	

Fasting insulin (mIU/ml)	GG	10,2394	4,98809	8,44-12,04	0,001
	
	GA	10,7138	4,53637	9,45-11,97	
	
	AA	21,5954	5,05475	8,87-34,32	

Homocysteine (μmol/L)	GG	2,3526	1,21	1,91-2,79	0,003
	
	GA	2,3925	1,18	2,06-2,72	
	
	AA	4.11	3,27	2,04-6,19	

Estradiol (pg/ml)	GG	29,57	17,069	22,36-36,78	0,226
	
	GA	40,74	33,594	29,69-51,78	
	
	AA	29,33	12,952	19,38-39,29	

DHEA-S (μg/dl)	GG	205,46	90,094	169,07-241,85	0,645
	
	GA	229,45	126,715	188,92-269,98	
	
	AA	202,78	112,325	116,44-289,12	

17 -OHP (ng/ml)	GG	1,878	0,846	1,543-2,212	0,906
	
	GA	1,835	1,2192	1,445-2,225	
	
	AA	1,709	0,8723	1,123-2,295	

Free-Testosterone (pg/ml)	GG	3,16	2,009	2,35-3,97	0,63
	
	GA	3,11	2,248	2,4-3,82	
	
	AA	3,8	1,982	2,47-5,13	

Total-Testosterone (ng/ml)	GG	1,278	3,0116	0,024-2,581	0,582
	
	GA	0,748	0,8182	0,458-1,039	
	
	AA	0,813	0,2997	0,562-1,063	

Prolactin (pmol)	GG	17,61	9,731	13,76-21,46	0,568
	
	GA	16,07	7,293	13,67-18,46	
	
	AA	18,8	6,125	14,42-23,18	

**Table 4:** Biochemical and Hormonal parameters between G2706A genotypes in PCOS patient group

	G2706A Genotype	Mean	SD	95% CI	p
BMI (k/m^2^)	GG	24,47	5,17	23,01-25,92	0,071
	
	GA	28,42	7,01	21,06-35,77	
	
	AA	20,63	1,25	18,64-22,61	

Fasting Serum Glucose (mg/dl)	GG	92,82	10,28	89,93-95,71	0,691
	
	GA	89,83	6,94	82,55-97,12	
	
	AA	90,00	4,32	83,13-96,87	

Fasting insulin (mIU/ml)	GG	12,5701	10,69	10,04-15,10	0.560
	
	GA	9,6271	4,12	7,25-12,00	
	
	AA	11,5217	4,24	8,82-14,22	

Homocysteine (μmol/L)	GG	2,7138	1,84	2,27-3,15	0.491
	
	GA	2,1653	0,97	1,60-2,73	
	
	AA	2,3987	1,21	1,63-3,17	

Estradiol (pg/ml)	GG	34,72	28,55	26,43-43,01	0,499
	
	GA	45,75	30,71	3,11-94,61	
	
	AA	50,50	47,90	25,72-126,72	

DHEA-S (μg/dl)	GG	216,10	101,88	186,52-245,69	0,079
	
	GA	302,67	156,34	138,60-466,74	
	
	AA	148,75	100,73	11,54-309,04	

17 -OHP (ng/ml)	GG	1,992	1,14	1,664-2,320	0,823
	
	GA	1,767	1,13	0,584-2,950	
	
	AA	1,725	0,60	0,767-2,683	

Prolactin (pmol)	GG	17,20	8,87	14,62-19,77	0,758
	
	GA	15,50	4,28	11,01-19,99	
	
	AA	14,50	3,51	8,91-20,09	

Free-Testosterone (pg/ml)	GG	3,49	2,48	2,77-4,20	0,839
	
	GA	3,92	1,48	2,36-5,47	
	
	AA	3,03	1,19	1,13-4,92	

Total-Testosterone (ng/ml)	GG	1,041	2,32	0,288-1,794	0,671
	
	GA	1,900	1,96	1,224-5,024	
	
	AA	,525	0,30	0,050-1,000	

### IV-Effects of ABCA genotypes on oxidative stress markers, homocystein levels in patients

In our study, the homocystein levels were significantly higher in PCOS patients with ABCA G1051A mutant genotype than those with heterozygote and wild genotypes (Table 3). The ABCA genotypes do not appear to have significant correlation with the plasma Hcy levels, MDA, NO and SH in PCOS patients.

## Discussion

Women with PCOS have multiple risk factors for the development of cardiovascular disease, including hyperandrogenemia, insulin resistance and glucose intolerance, obesity, and central fat deposition [18]. Women with PCOS are also at increased risk for the development of the metabolic syndrome [19,20].

Additionally, different markers of clinical and subclinical atherosclerosis, including serum markers (for example fibrinogen, high sensitive C-reactive protein and homocysteine, oxidative stress markers, PAI-1, TPA), carotid intimae-media thickness, and echocardiographic findings have also been found to be changed [21-25]. In our study, no meaningful correlation between homocystein, NO MDA, and SH levels between patient and control groups. Fibrinogen levels were found to be statistically higher in PCOS patients group than the controls. However, homocystein levels were significantly higher in PCOS patients with ABCA G1051A mutant genotype than those with heterozygote and wild genotypes.

Women with PCOS have a higher prevalence and a greater degree of hyperinsulinemia, and insulin resistance than weight-matched control subjects [26-28]. However, in our study, fasting insulin levels were not different from the controls when compared to healthy controls.

Dyslipidemia is a common metabolic abnormality in PCOS, although the reported types and extent of lipid aberrations have been variable [29,30]. PCOS women have been demonstrated to have substantially higher TC and LDL levels than control women < 45-years-old, after adjustment for BMI and hyperinsulinemia [31].

IR represents another key factor implicated in the dyslipidemia of PCOS. Glueck et al. reported that 46% of women with PCOS suffered from the metabolic syndrome [32]. Within this subgroup of women, lipid abnormalities were found to be extremely common. 95% of these women demonstrated decreased levels of high-density-lipoprotein (HDL), whereas 56% had hypertriglyceridemia. In this study, no difference was determined in PCOS patient group in terms of HDL cholesterol when compared to the controls, while TG values were determined to be statistically meaningfully high in patient group. In this study, LDL cholesterol and total cholesterol levels were found to be statistically meaningfully high in PCOS patient group when compared to the controls.

IR and compensatory hyperinsulinemia are considered to be main responsible for the lipidemic aberrations of obesity and PCOS. Besides, genetic factors may also contribute to the formation of dyslipidemia in PCOS disease [33]. Members of the ATP-binding cassette (ABC) transporter family, such as ABCA1, have been shown to control cellular lipid metabolism [34,35]. Changes happened in ATP binding cassette transporter 1 (ABC1) gene encoding a protein regulating entry and exit from cell membrane may contribute to dyslipidemia in patients with PCOS.

Jerome I. Rotter et al. found a relation between ABCA1 G1051A polymorphism and HDL-cholesterol elevation. This relation conflicts with previous studies. Again, these researchers determined a correlation between ABCA1 G1051A gene polymorphism and moderate LDL-cholesterol lowness [36,37]. In our study, the genotype ABCA G1051A distribution didn't differ between control and PCOS groups. However, the distribution of ABCA G2706A genotype differed between the control group and the PCOS patients. Frikke-Schmidt et al. determined higher G2706A gene polymorphism allele in patient group with low HDL cholesterol [38]. In our study, the frequency of the polymorphic A allele of ABCA G2706A gene was higher in PCOS patients than control group.

There was no statistically significant difference in PCOS patients between ABCA G1051A genotypes (AA, GA and GG) and BMI, fasting insulin, fasting glucose, triglyceride levels, HDL levels, LDL levels, fasting blood glucose levels, f-testosterone, fibrinogen and 17-OHP levels. However, in PCOS patients, fasting insulin levels and homocystein were found to be significantly higher in ABCA G1051A mutant genotype than heterozygote and wild genotypes.

In conclusion, we found AA genotype and A allele of ABCA G2706A in PCOS patients. The fasting insulin and homocystein levels were significantly higher in PCOS patients with ABCA G1051A mutant genotype than those with heterozygote and wild genotypes. However, it is determined that ABCA1 G2706A and ABCA G1051A polymorphisms have no effect on lipid oxidative stress parameters in patients. Future studies should explain the specific roles of other genes (e.g CETP, LDL receptor and hepatic lipase) in the pathophysiology of dyslipidemias in PCOS.

## Competing interests

The authors declare that they have no competing interests.

## Authors' contributions

In this study, MK, VC, AT carried out the molecular genetic studies, SC, FS, AGO, CY conceived of the study, and participated in its design and coordination. AZ, MY, participated in the sequence alignment and drafted the manuscript. MK, ME participated in the design of the study and performed the statistical analysis and participated in the sequence alignment. MO, carried out the immunoassays. All authors read and approved the final manuscript.
